# Analysis of coronary angiography related psychophysiological responses

**DOI:** 10.1186/1475-925X-10-71

**Published:** 2011-08-11

**Authors:** Şükrü Okkesim, Sadık Kara, Mehmet G Kaya, Musa H Asyali

**Affiliations:** 1 Institute of Biomedical Engineering, Fatih University, Buyukcekmece, Istanbul, 34500, Turkey; 2 Department of Cardiology, Erciyes University, Talas, Kayseri, 38039, Turkey; 3 Faculty of Engineering and Architecture, Abdullah Gül University, Kocasinan, Kayseri, 38090, Turkey; 4 Department of Electrical and Electronics Engineering, Zirve University, Kizilhisar Campus, Gaziantep, 27260, Turkey

## Abstract

**Background:**

Coronary angiography is an important tool in diagnosis of cardiovascular diseases. However, it is the administration is relatively stressful and emotionally traumatic for the subjects. The aim of this study is to evaluate psychophysiological responses induced by the coronary angiography instead of subjective methods such as a questionnaire. We have also evaluated the influence of the tranquilizer on the psychophysiological responses.

**Methods:**

Electrocardiography (ECG), Blood Volume Pulse (BVP), and Galvanic Skin Response (GSR) of 34 patients who underwent coronary angiography operation were recorded. Recordings were done at three phases: "1 hour before," "during," and "1 hour after" the coronary angiography test. Total of 5 features obtained from the physiological signals were compared across these three phases. Sixteen of the patients were administered 5 mg of a tranquilizer (Diazepam) before the operation and remaining 18 were not.

**Results:**

Our results indicate that there is a strong correlation between features (LF/HF, Bk, DN1/DN2, skin conductance level and seg_mean) in terms of reflecting psychophysiological responses. However only DN1/DN2 feature has statistically significant differences between angiography phases (for diazepam: p = 0.0201, for non_diazepam p = 0.0224). We also note that there are statistically significant differences between the diazepam and non-diazepam groups for seg_mean features in "before", "during" and "after" phases (p = 0.0156, 0.0282, and 0.0443, respectively).

**Conclusions:**

The most intense sympathetic activity is observed in the "during" angiography phase for both of the groups. The obtained features can be used in some clinical studies where generation of the customized/individual diagnoses styles and quantitative evaluation of psychophysiological responses is necessary.

## Background

Many diseases are associated with stress and anxiety, and it is well known that stress is a strong contributing factor in the development of some important conditions like heart and cerebrovascular diseases, hypertension, and peptic ulcer [1-3]. Conversely, the role of "being in a good mood" in reducing the risk of disease, shortening the treatment period, and strengthening the immune system has been shown in the literature [4]. Although psychophysiological changes have great influence on people, stress and anxiety do not have a standardized tabulation or scoring system for their quantitative or objective assessment.

Invasive tests like coronary angiography cause anxiety, fear, and stress that lead to a decrease in quality of life while awaiting the procedure and an increase in complication rates during and after the test [5-9].

Coronary angiography is the most common medical test for the diagnosis of cardiovascular diseases, which are the major cause of death in the developed countries and in many developing countries. Coronary angiography test is carried out on more than 1 million people in the United States in 1993 and it is estimated that this number reached 3 million in 2010 [10,11].

The risk of major complications during angiography, like perforation of the coronary vessels, low blood pressure due to severe allergic reaction, and rhythm disturbances that would require temporary cardiac pacing are rarely seen (< 2%) [10]. Other than its physical effects, angiography is also a psychologically important event and most patients find coronary angiography to be extremely stressful [6,12,13].

In order to evaluate anxiety, fear or stress during angiography, questionnaires are filled while patients were on the operation table in the catheterization laboratory. Using Visual Analogy Scale (VAS), Hospital Anxiety Depression (HAD), and State Trait Anxiety Inventory (STAI), Heikkila *et al. *reported that 80% of the 243 patients had anxiety because of coronary angiography [5]. Mott carried out a similar study on 30 patients using STAI test and concluded that coronary angiography causes anxiety and psychological preparation is beneficial in reducing patients' anxiety before cardiac catheterization [6]. Same findings were obtained on 60 adult patients scheduled for cardiac catheterization by Anderson and Masur [7]. Caldwell *et al. *investigated fears and beliefs of patients regarding cardiac catheterization. They asked questions like 'Can you tell me more about that issue/fear/concern/belief' to 20 patients and concluded that cardiac catheterization causes fear because of lack of control about physical and psychosocial aspects of the procedure, unknown test results, and possible medical complications [8]. De-Jong-Watt and Arthur investigated the impact of waiting for first-time coronary angiography (CA) on patients' anxiety and health-related quality of life. They used Seattle Angina Questionnaire (SAQ) and a direct-estimation, 4-point Likert scale to measure health-related quality of life and perceived anxiety. According to their results, waiting for CA may have a negative impact on anxiety level and health-related quality of life [9].

These studies showed that invasive tests like coronary angiography cause fear, stress, and anxiety. However, throughout these studies mostly questionnaires were used to measure fear, stress, or anxiety, and therefore only subjective or individual-dependent results were obtained. In some cases, subjects may not identify and/or express their own feelings or they may not want to share their private information with others [14,15]. Based on finding of these studies, further studies were carried out to investigate how fear, stress or anxiety could be reduced on patients who undergo an invasive test in the hospital. They used guided imagery techniques, music and progressive muscle relaxation to reduce stres and anxiety [6, 13 and 15]. On the other hand diazepam is the accepted method in the clinical world to deescalate anxiety and used widely [16]. If the tranquilizer effect of the diazepam can be evaluated using physiological responses, the effectiveness of the diazepam and other methods (guided imagery techniques, music etc.) can be compare and discuss quantitatively.

The above literature survey reveals that the investigation of psychophysiological responses due to coronary angiography has not been dealt with objective methods and only subjective or individual-dependent results can be obtained from those studies.

Therefore, in this study we want to investigate psychophysiological responses to coronary angiography (CA) to bring out whether psychological changes originated from CA can be evaluated using measurable values e.g. physiological responses. More specifically, we would like to use physiological signals and their appropriate features to measure and evaluate angiography related psychophysiological responses in an objective manner. One of these benefits will be the quantitative assessment of the psychological conditions of the patients who will undergo invasive operations. With the help such knowledge, invasive operations will be performed more effectively, the health of the patients will be risked less, customized treatment styles may be performed in the future and new methods may be developed in the diagnosis and treatment of depressive disorders like depression that have direct influence on human health.

The studies conducted since Canon [17], who introduced the fight-flight concept in his study, have demonstrated that when a stress source is encountered, Sympathetic Nervous System (SNS) is stimulated and consequently changes occur in the physiological parameters controlled by the SNS. In our study, two different groups were gathered: One group was administered with diazepam and the other was not. Diazepam is a commonly used drug for treating anxiety, insomnia and muscle spasms. It is also used before some painful medical procedures to deescalate anxiety, and in some surgical operation to cause amnesia [16]. In this way, we considered that differences between the non-diazepam group and the diazepam group in which SNS was suppressed via diazepam may be evaluated statistically.

As physiological signals reflecting psychophysiological responses, we have recorded Electrocardiography (ECG), Blood Volume Pulse (BVP), and Galvanic Skin Response (GSR) from 34 patients who have undergone coronary angiography.

Sympathetic and parasympathetic nervous systems control the heart rate. SNS tends to increase heart rate and its response is slow. Parasympathetic nervous system (PNS), on the other hand, tends to decrease heart rate and mediates faster [18,19]. Therefore, Heart Rate Variability (HRV) could be used in quantitative assessment of stress and anxiety. Frequency domain analysis of HRV was performed and the Power Spectral Density (PSD) for low frequency (*LF*: 0.04-0.15 Hz) and high frequency (*HF*: 0.15-0.40 Hz) bands were computed. The *HF *and *LF *components of the HRV are controlled by PNS and SNS respectively [18,19]. Thus the *LF/HF *ratio gives an index of autonomic balance between sympathetic and parasympathetic activities.

The vasomotor activity, controlling the wall width of the veins is controlled by SNS. Therefore, the alterations in the BVP signal amplitude gives information about the stimulation of SNS. An increase in the BVP signal amplitude shows that there is a decrease in SNS stimulation, which leads to widening of vein walls and more blood circulation.

The moment the left ventricle pumps blood to the body, the BVP signal amplitude is at the maximum. In some cases, a second visible increase may occur in the descending margin of this signal. The notch, formed by the closing of the valves belonging to the aorta is called "Dicrotic Notch". When a stimulus that would increase heart beat pressure is present, left ventricular contraction pressure overcomes the negative effect formed due to the aorta valves and dicrotic notch cannot be seen in the BVP signal [20]. Correspondingly, the power of the second harmonic, which corresponds to the dicrotic notch, decreases in the frequency spectrum [21].

The activity of sweat glands is also controlled by SNS. When a person is scared or stimulated by an anxiety rising stimulus, the activity of the sweat glands increase till the glands reach saturation and a rapid increase in the *skin conductivity *or GSR occurs. Therefore, the change seen in the skin conductivity reflects the change in the stimulation level of SNS [22,14].

The recordings were repeated at three phases or stages: "1 hour before," "during," and "1 hour after" the coronary angiography test. The recorded signals were then processed for feature extraction and obtained features were analyzed statistically across groups (diazepam, non-diazepam) and different angiography phases.

## Methods

### Measurement of Physiological Signals

ECG, BVP, and GSR signals were recorded from 34 patients consisting of 22 males and 12 females (Mean Age: 59.6; Standard Deviation: 11.5). These patients were randomly selected among the admitted patients for CA at Cardiology Center of Erciyes University Hospital, Kayseri, Turkey in December 2008. There were no age or gender limitations. The medical part of the study was designed by a cardiologist in our research team. In that design, patients were assigned to the diazepam or non-diazepam groups randomly. All the patients have undergone coronary angiography operation at the catheter laboratory of the Cardiology Center of Erciyes University. Prior to the study, informed consent of the patients and approval of the Erciyes University Ethical Committee was obtained. The ECG, BVP, and GSR recordings were done at three phases as "1 hour before," "during," and "1 hour after" the angiography test for a duration of at least 5 minutes. Sixteen of the patients were administered 5 mg of diazepam just before the angiography. Except for the diazepam, they were not administered any other medications that may affect the ANS. The MP150 biomedical data acquisition unit (Biopac Systems, Goleta, CA, USA) was used for recording signals. All the signals were sampled at 1000 Hz and digitized (A/D converted) at a resolution of 12 bits per sample by the MP150 unit.

The ECG is one of the best known biomedical signals in psychophysiological research and serves as the most accurate measure of cardiac function. As such, it is the basis for many studies including Heart Rate Variability (HRV) analysis [18]. The ECG100C amplifier module of the MP150 unit was used to amplify and filter with the following settings: 35 Hz Low-Pass (LP) filter, 0.05 Hz High-Pass (HP) filter, and 500 gain. In order to reduce electrode impedance, skin surface was abraded and electrode gel was applied before electrode placement.

Photo-plethysmograph is a method for detecting blood volume in peripheral vessels. A Light Emitting Diode (LED) placed at a fingertip sends an infrared light beam and a photo-detector placed next to the LED receives reflected light from the tissues underneath. With each contraction of the heart, blood is pumped to the vascular system so that the vessels including the capillaries under the LED are filled with blood, and the amount of light reaching photo-detector changes. The resultant nearly periodic signal is referred to as the BVP signal [23]. BVP signals were recorded using PPG 100C amplifier and TSD200 transducer modules of the MP150 unit. The probe of the TSD200 module, which operates on 860 ± 60 nm wave length, was placed on the ring finger. The module filter settings were as 3 Hz LP filter and 0.5 Hz HP filter.

The GSR, also known as the electro-dermal activity, is the signal obtained by measurement of skin conductivity by passing a small current (10 μA/cm^2^) between the two electrodes placed at the fingers [22]. GSR signals were recorded using GSR100C amplifier and TSD203 transducer modules of the MP150 unit in DC mode. The 6 mm diameter Ag-AgCl electrodes were filled with conductive gel and placed on the middle finger and forefinger of the non-dominant hand. The GSR signal is directly influenced by the condition of skin surface where electrode is in contact. Different parts of skin may show different resistance change due to stress related sweating. In order to minimize the variance that may arise due to this effect, recordings were taken from the non-dominant hands of the subjects.

**Figure 1:**
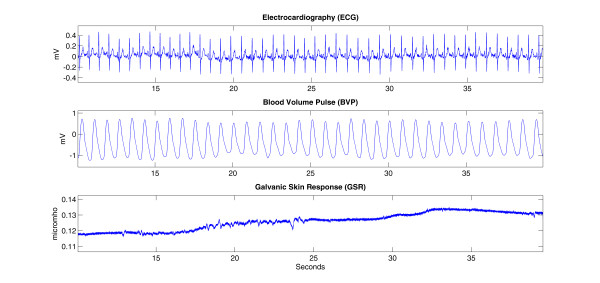
**The raw ECG, BVP and GSR signals recorded from a patient in the non-diazepam group during the "before angiography" phase**.

Figure 1 shows some sample raw signals obtained from a non-diazepam subject during the "before angiography" phase.

### Analysis of Physiological Signals

The segment of the ECG waveform that corresponds to ventricular depolarization is named as QRS complex. The QRS complex can easily be identified on normal ECG due to its relatively large acceleration and amplitude deflection, compared to other parts of the ECG. Since the R point of the QRS complex marks the peak of ventricular depolarization, the beat instants are taken at these points and consequently beat-to-beat intervals are determined as the length in time from one R wave to the next one. These intervals are called RR or inter beat intervals. While carrying out the HRV analysis, we have followed the methodological guidelines listed in [18]. To detect R peaks, we applied the algorithm suggested by Manriquez and Zhang [24,25]. RR interval series is inherently nonuniformly sampled series. Interpolation is necessary to produce a uniformly sampled HRV time series out of an RR interval series. The interpolation frequency was chosen as 4 Hz [26]. After interpolation, PSD analysis was performed using Welch's method with Hann window.

The Welch method calculates an average over the modified periodograms. Periodogram is defined as a classical nonparametric method to obtain PSD. The main drawback of the periodogram method is the effect of offside lobe leakage due to finite data sets. To minimize the effect of the offside lobe leakage, time series of the signal is divided into overlapping sequences then each data sequence is windowed in order to smooth the edges of the signals [27,28]. Due to this improvement Welch method was used to estimate the power spectral density of the interpolated HRV signal.

n = 0,1, ..., (L-1),

i = 0,1, ..., (K-1),(1)

The *i*th modified periodogram is:(2)

where U is the normalizing constant.(3)

The power spectral density is:(4)

L is the length of the interpolated HRV signal and W(n) is the Hann function [27].

**Figure 2:**
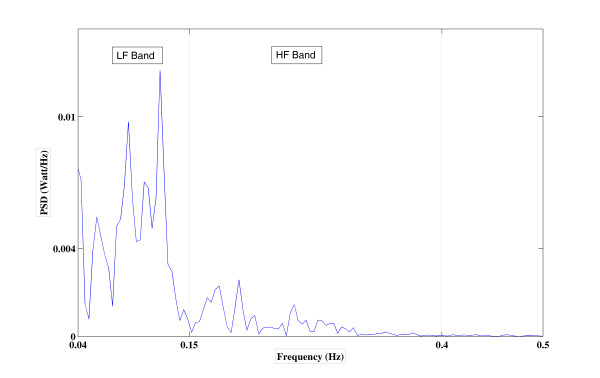
**Power spectral density analysis of HRV signal of a patient in the non-diazepam group "during the angiography" phase**.

In our case, the window size of 256 samples and 50% overlap were chosen so that 0.0156 Hz spectral resolution was achieved (Figure 2). In this way, power in *LF *and *HF *bands and their ratio, *LF/HF*, were calculated for each patient.

**Figure 3:**
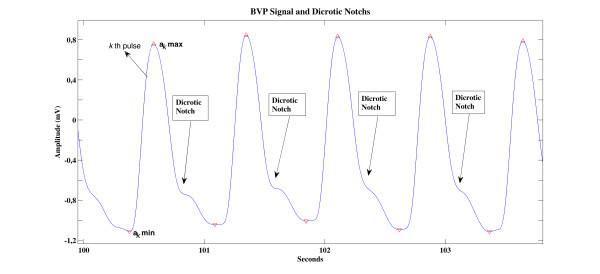
**Local maximum and minimum points in the BVP signal and dicrotic notches**.

In the analysis of the BVP signal, we first find local maximum and minimum points of the signal. In Figure 3, we present a sample BVP signal with dicrotic notchs and the peaks detected on this signal. Defining *a_k_*max and *a_k_*min as the local maximum and minimum amplitude values of the *k*th pulse in the BVP signal, a series of pulse amplitude values are obtained as:(5)

As BVP signal features to be compared across different phases of the angiography, we use the median value of these pulse amplitude series obtained from each subject.

**Figure 4:**
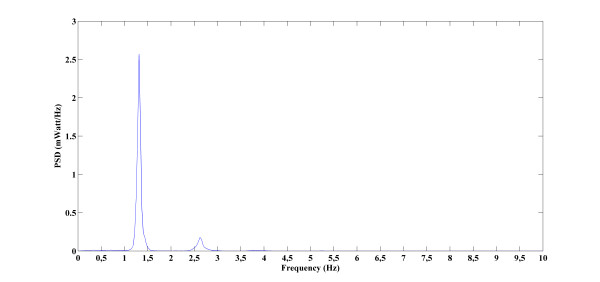
**Power spectral density analysis of a patient's BVP signal in the non-diazepam group "during the angiography" phase**. (The duration of the signal and sampling frequency was respectively 467,407 second and 1000Hz).

The PSD analysis was also performed on each subject's BVP signal to obtain the amplitudes of 1^st ^and 2^nd ^harmonics. Along with these two features, the ratio of these first two harmonics was also calculated as a third BVP signal feature to be used in the statistical comparisons. BVP signals PSDs were computing using the Welch method (Eq.s 1, 2, and 3). The Hann windowing was used and analysis window length was taken as one twentieth of the length of the data. Figure 4 shows the PSD of a sample BVP signal.

In GSR studies in which signal recording is conducted during the sudden stimulus-free period (tonic skin conductance level), the most common feature is the mean amplitude value of the signal and is named as skin conductance level [22]. As features to be used in GSR analysis, *mean *amplitude values of the signals were calculated for each patient at each angiography stage. In addition, the vector forming GSR signal was divided into 1 second long segments, and mean values for each of these segments were also calculated:(6)

Here, *t*_min _and *t*_max _are given as:(7)(8)

where, *f_s _*is the sampling frequency, *k = *1, 2, 3,...,*N/f*, and is *N *the length of the GSR signal.

Since we had two groups of subjects (diazepam and non-diazepam) and three different angiography phases to be analyzed (before, during, and after), we have carried out statistical comparisons of feature values across both dimensions. We used one way ANOVA with a 95% confidence limit and a p-value of less than 0.05 was considered as statistically significant. Bonferroni type of critical value was used for multiple comparisons to find out not just whether there are any differences among the means, but particularly which pairs of means are significantly different.

All the data processing and statistical analyzing was carried out using in-house programs developed under MATLAB R2009b Software (MathWorks Inc., Natick MA, USA).

## Results

In this study, in order to evaluate the psychophysiological responses that are caused by coronary angiography, an invasive process that is used for the diagnosis of atherosclerosis, the ECG, BVP and GSR signals were recorded at different stages of the operation and some features that could reflect the changes in the sympathetic nervous system were calculated and analyzed. In addition, the influence of diazepam, a tranquilizer drug which is typically administered during angiography operations, on psychophysiological responses is also investigated. To this end, 16 of the 34 patients participating in the study were administered diazepam and 18 were not. Detailed demographic data of the patients is reported in Table 1.

**Table 1:** Subject demographics

	Diazepam Group	Non-Diazepam Group
Number of Subjects	16	18

Age (Mean ± Standard Deviation)	60 ± 12	59 ± 11

Gender (Male/Female)	9/7	13/5

Education Level	Primary School: 15 High School: 1 University: 0	Primary School: 15 High School: 2 University: 1

Groups mean and standard error of the mean (SEM) values were calculated for the each feature and line graphs with angiography phase on the x-axis were figured using group mean and SEM to provide a comparison. Groups mean and SEM. were pointed on the graph as Mean ± SEM.

In the tables, we present the p-values for both "across angiography phase" and "across group" comparisons with an estimate of the difference in group means and a confidence interval.

In the analysis of HRV signal, the *LF*/*HF *ratio was used as a feature. An increase in this ratio corresponds to imbalance of activity in the nervous system. In the table 2 and 3, we present the p-values with an estimate of the difference in group means and a confidence interval for both "across angiography phase" and "across group" comparisons.

**Table 2:** Results of the statistical analysis of the LF/HF ratio for phase comparison

			Across Phase Comparison	
		
		The Estimated Difference in Means	Confidence Interval For the True Mean	P-Value
Diazepam	Before-During	-0.5989	[-3.4960, 2.2982]	0.8563
		
	Before-After	-0.0807	[-2.9778, 2.8164]	
		
	During After	0.5183	[-2.3789, 3.4154]	

Non_Diazepam	Before-During	-0.8818	[-2.4851, 0.7214]	0.4003
		
	Before-After	-0.3832	[-1.9865, 1.2201]	
		
	During After	0.4986	[-1.1047, 2.1019]	

**Table 3:** Results of the statistical analysis of the LF/HF ratio for group comparison

	Across Group Comparison (Diazepam - Non_diazepam)		
	
	The Estimated Difference in Means	Confidence Interval For the True Mean	P-Value
Before	**1.3845**	**[0.1586, 2.6103]**	**0.0281**

During	1.1016	[-1.4706, 3.6738]	0.3895

After	1.0820	[-0.4370, 2.6010]	0.1565

**Figure 5:**
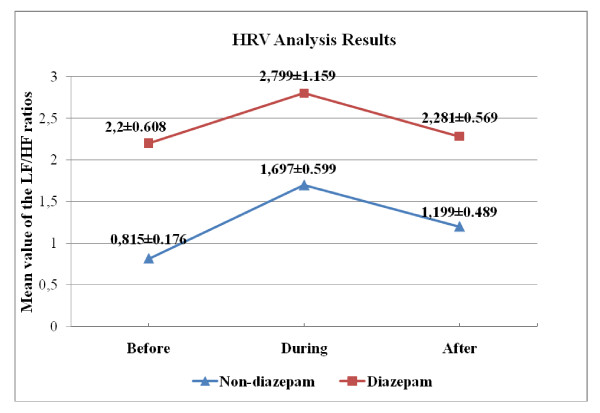
**Over-all LF/HF ratios (Mean ± SEM) across different angiography phases**.

In reference to tables 2, and 3 for both diazepam and non-diazepam groups, we do not observe any statistically significant change across different angiography phases. As shown in Figure 5, the LF/HF is relatively higher at the "during angiography" phase for both groups, which confirms that there is an increased SNS activity during the operation. We notice that the SEM has the largest value in the "during angiography" phase for both groups. In other words, deviation for the "during angiography" phase is more than the other situations. One possible reason of this is extrasystoles due to the catheterization. Another reason may be the different recording duration of the signals. Recording duration could not be fixed due to the angiography operation. All the recordings were done for a duration of at least 5 minutes and PSD analysis were performed for the whole duration of the HRV signals to minimize the effect of the different recording duration [18].

On the other hand, a different feature that will be a more standard index or criteria can be computed like organization index (OI) [29]. If such a feature will be used as an indicator, physiologist must find out how the psychophysiological response is related to the feature, regarding the physiological meanings.

We also note that LF/HF ratio is elevated in the diazepam group at all angiography phases. Further, at the "before angiography" phase there is a statistically significant difference (p = 0.0281) diazepam and non-diazepam groups. Interpretation of these observations is relatively cumbersome, as one would normally expect a fall in the sympathetic activity with the action/influence of diazepam and consequently a lower LF/HF ratio in the diazepam group. However, when we refer to recent literature in order to check the influence of diazepam on HRV (especially on LF/HF ratio) we come across studies [30,31] confirming our relatively interesting results. For instance, the study in [30] concludes that administration of diazepam did not cause a significant change especially in elderly. Among different reasons explanations for this phenomenon T. Kitajima, T. Kanbayashi Y. Saito et al. [31] argues "One possible explanation is that the response of the cardiac sympathetic nerve to diazepam is dissociated with muscle sympathetic nerve activity, which modulates peripheral vascular resistance, but not the heart rate itself." Therefore, we conclude/observe that HRV is not a good indicator to evaluate the influence of the tranquilizer on the psychophysiological responses.

**Figure 6:**
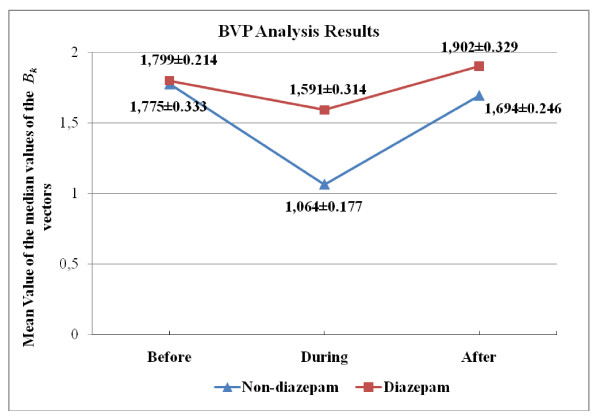
**Over-all median *B_k _*values (Mean ± SEM) across different angiography phases**.

When sympathetic activity increases, vein walls narrow and the volume of blood in the veins decreases and consequently the amount of infrared light reflected from the veins and BVP amplitude decreases. When the sympathetic activity decreases, the contrary occurs. Therefore, the amplitude of BVP signal is inversely proportional to SNS activity. In this case, since the distribution of BVP amplitude values, namely *B_k_*'s in Eq.4, is not necessarily symmetric, therefore we have used *median *instead of *mean*, as a measure of central tendency. This way, we have also lumped (summarized) many BVP amplitude values obtained from a subject during an angiography phase into a single figure. In the table 4 and 5, we present the p-values with an estimate of the difference in group means and a confidence interval for both "across angiography phase" and "across group" comparisons. Variation of the group mean of the B_*k *_medians according to the angiography phase is seen in Figure 6.

**Table 4:** Results of the Statistical analysis of the B_*k *_for phase comparison

		Across Phase Comparison		
		
		The Estimated Difference in Means	Confidence Interval For the True Mean	P-Value
Diazepam	Before-During	0.2077	[-0.8128, 1.2281]	0.7433
		
	Before-After	-0.1037	[-1.1241, 0.9168]	
		
	During After	-0.3114	[-1.3318, 0.7091]	

Non_Diazepam	Before-During	0.7110	[-0.1991, 1.6211]	0.1166
		
	Before-After	0.0809	[-0.8292, 0.9910]	
		
	During After	-0.6301	[-1.5402, 0.2800]	

**Table 5:** Results of the Statistical analysis of the B_*k *_for group comparison

	Across Group Comparison (Diazepam - Non_diazepam)		
	
	The Estimated Difference in Means	Confidence Interval For the True Mean	P-Value
Before	0.0240	[-0.8046, 0.8525]	0.9534

During	0.5273	[-0.1863, 1.2409]	0.1421

After	0.2086	[-0.6171, 1.0343]	0.6104

We notice that the mean BVP amplitude decreases during the angiography operation for both groups (Figure 6). This is expected, as increased SNS activity due to the operation reduces BVP amplitude. Results of the across phase and group comparison indicate that there is no statistically significant difference among different phases and groups (Tables 4 and 5). We conclude that BVP amplitude value is not a good indicator to evaluate psychophysiological responses that the subjects undergo during coronary angiography.

**Figure 7:**
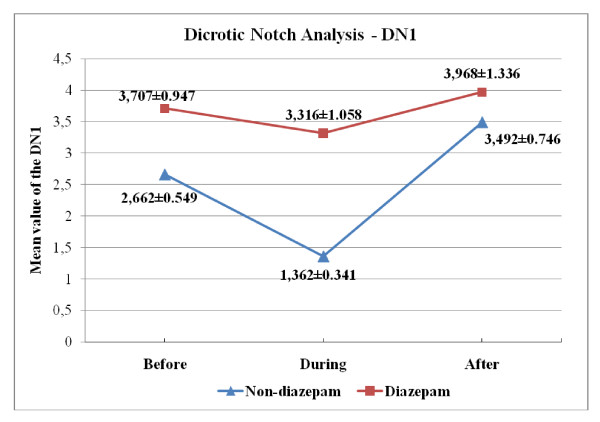
**Over-all DN1 values (Mean ± SEM) across different angiography phases**.

**Figure 8:**
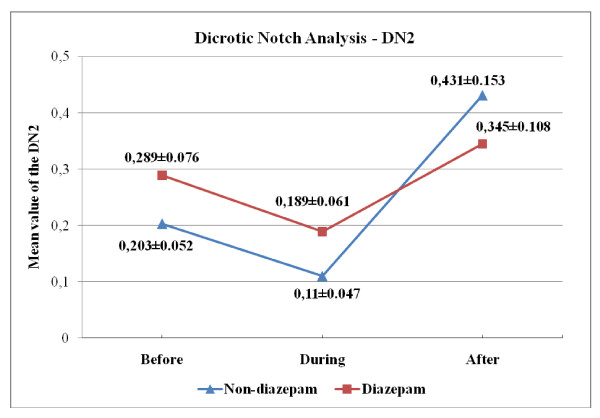
**Over-all DN2 values (Mean ± SEM) across different angiography phases**.

**Figure 9:**
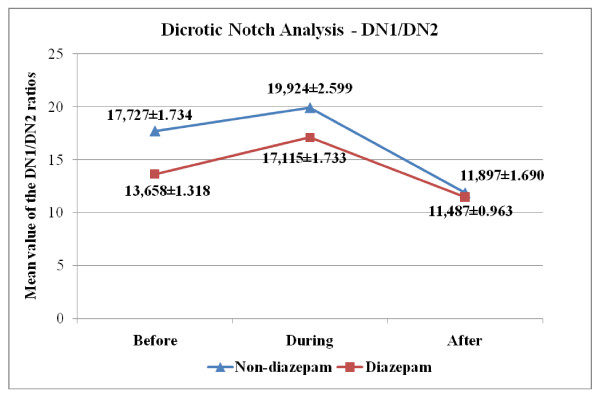
**Over-all DN1/DN2 values (Mean ± SEM) across different angiography phases**.

Another set of features obtained from the BVP signal is the "dicrotic notch." When the spectrum of BVP signal is examined, along with the first/fundamental harmonic that is due to the periodic nature of the BVP signal, a second peak that is associated with the presence of dicrotic notch is noticed [20,21]. In the study, peak values of first (DN1) and second (DN2) harmonics were obtained from the PSD of BVP signal. In the table 6 and 7, we present the p-values with an estimate of the difference in group means and a confidence interval for both "across angiography phase" and "across group" comparisons for DN1/DN2 values. Variation of the group mean of the DN1, DN2 and DN1/DN2 values according to the angiography phase is seen in Figure 7, 8 and 9, respectively.

**Table 6:** Results of the Statistical analysis of the DN1/DN2 for phase comparison

		Across Phase Comparison		
		
		The Estimated Difference in Means	Confidence Interval For the True Mean	P-Value
Diazepam	Before-During	-3.4570	[-8.2900, 1.3760]	**0.0201**
		
	Before-After	2.1710	[-2.6620, 7.0040]	
		
	During After	**5.6280**	**[0.7950, 10.4610]**	

Non_Diazepam	Before-During	-2.1975	[-9.3755, 4.9805]	**0.0224**
		
	Before-After	5.8302	[-1.3478, 13.0082]	
		
	During After	**8.0277**	**[0.8497, 15.2057]**	

**Table 7:** Results of the Statistical analysis of the DN1/DN2 for group comparison

	Across Group Comparison (Diazepam - Non_diazepam)		
	
	The Estimated Difference in Means	Confidence Interval For the True Mean	P-Value
Before	-4.0692	[-8.5906, 0.4522]	0.0761

During	-2.8097	[-9.3415, 3.7221]	0.3874

After	-0.4100	[-4.5061, 3.6861]	0.8397

As seen in Figure 9, the mean of the DN1/DN2 is relatively higher at the "during angiography" phase for both groups, which confirms that there is an increased SNS activity during the operation. Looking at the mean DN1 and DN2 values in the two groups, we also note that the decrease in the DN2 values cause an increase in the DN1/DN2 values for "during angiography" phase and a dramatic increase in the DN2 values cause a decrease in the DN1/DN2 values for the "after angiography" phase (Figure 7, 8). Upon reviewing table 6 and 7, we notice that the mean of "during angiography" phase minus the mean of "after angiography" phase is estimated to be 5.6280, and a 95% confidence interval for this difference is [0.7950, 10.4610] for diazepam group. And for the non-diazepam group the estimated difference in means is 8.0277 and the confidence interval is [0.8497, 15.2057]. Therefore, we can conclude that the during angiography phase's DN1/DN2 values and after angiography phase's DN1/DN2 values are different, statistically.

Actually, hospitalization and the waiting period for an invasive test or surgery is difficult for all the patients and some research showed that waiting for surgery impairs the quality of life for patients [32]. Anxiety and fear caused by waiting for angiography, increased values but not as much as the process itself. Therefore, it is concluded that the anxiety and fear of the person increase before angiography and feels most comfortable after angiography compared to other phases. p-values and confidence interval confirm our interesting results.

These findings support the anticipation that when a person encounters a stressful situation, the left ventricular contraction pressure overcomes the negative effect due to the aorta valves and as a result the power of the second harmonic (DN2) decreases. Therefore, we conclude that DN1/DN2 ratio is a feature that has the potential to properly reflect the psychophysiological responses.

**Figure 10:**
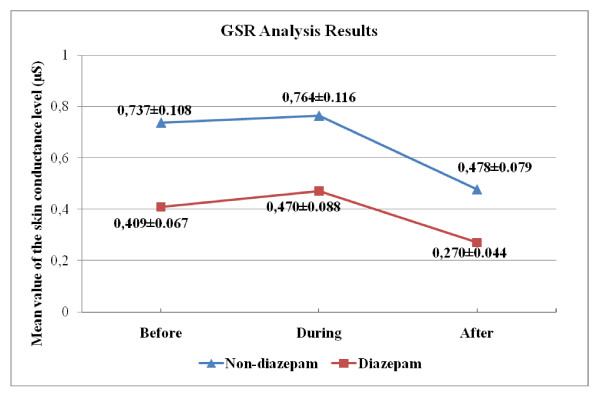
**Over-all mean GSR values (Mean ± SEM) across different angiography phases**.

The most common feature used in GSR analysis is the mean amplitude value of the signal, namely "skin conductance level" [22]. In the table 8 and 9, we present the p-values with an estimate of the difference in group means and a confidence interval for both "across angiography phase" and "across group" comparisons for skin conductance level. Variation of the group mean of the skin conductance level according to the angiography phase is seen in Figure 10.

**Table 8:** Results of the statistical analysis of the GSR signals for phase comparison

		Across Phase Comparison		
		
		The Estimated Difference in Means	Confidence Interval For the True Mean	P-Value
Diazepam	Before-During	-0.0608	[-0.3026, 0.1810]	0.1222
		
	Before-After	0.1384	[-0.1035, 0.3802]	
		
	During After	0.1992	[-0.0426, 0.4410]	

Non_Diazepam	Before-During	-0.0270	[-0.3843, 0.3303]	0.0998
		
	Before-After	0.2601	[-0.0973, 0.6174]	
		
	During After	0.2871	[-0.0703, 0.6444]	

**Table 9:** Results of the statistical analysis of the GSR signals for group comparison

	Across Group Comparison (Diazepam - Non_diazepam)		
	
	The Estimated Difference in Means	Confidence Interval For the True Mean	P-Value
Before	**-0.3281**	**[-0.5944, -0.0619]**	**0.0173**

During	-0.2943	[-0.5960, 0.0074]	0.0556

After	**-0.2064**	**[-0.3977, -0.0152]**	**0.0353**

We find out that the mean skin conductance obtains its highest value during the angiography operation for both groups (Figures 10). This is expected, as increased SNS activity due to the operation increases GSR amplitude. The mean skin conductance for "before angiography" phase is smaller than the mean skin conductance of "during angiography" phase. On the other hand, the difference between them was much less compared to a difference between "during" and "after". The mean skin conductance for "after angiography" is the smallest value of three.

This observation is in-line with the finding obtained for the DN1/DN2, which confirms that waiting for angiography cause anxiety and fear.

Results of the across phase comparison indicate that there are no statistically significant difference among different phases in both of the groups (Table 8). We also note that there are statistically significant differences between diazepam and non-diazepam group for skin conductance level in the "before" and "after" angiography phases (Table 9).

**Figure 11:**
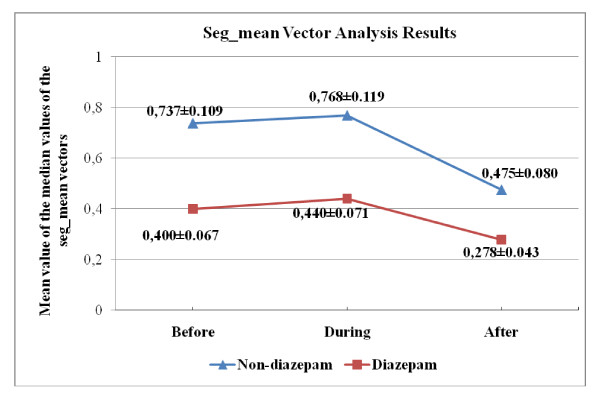
**Over-all median seg_mean values (Mean ± SEM) across different angiography phases**.

In addition, as mentioned above, the vector forming GSR signal was first divided into 1-second long segments, and the mean values for each of these segments (seg_means) were calculated (Eq.s 5 through 7). Next, median of these seg_mean values were computed. In the table 10 and 11, we present the p-values with an estimate of the difference in group means and a confidence interval for both "across angiography phase" and "across group" comparisons for seg_mean median values. Variation of the group mean of the seg_mean median values according to the angiography phase is seen in Figure 11.

**Table 10:** Results of the statistical analysis of the seg_mean for phase comparison

		Across Phase Comparison		
		
		The Estimated Difference in Means	Confidence Interval For the True Mean	P-Value
Diazepam	Before-During	-0.0407	[-0.2581, 0.1767]	0.1686
		
	Before-After	0.1211	[-0.0963, 0.3385]	
		
	During-After	0.1618	[-0.0557, 0.3792]	

Non_Diazepam	Before-During	-0.0310	[-0.3940, 0.3321]	0.0999
		
	Before-After	0.2621	[-0.1010, 0.6252]	
		
	During-After	0.2931	[-0.0700, 0.6561]	

**Table 11:** Results of the statistical analysis of the seg_mean for group comparison

	Across Group Comparison (Diazepam - Non_diazepam)		
	
	The Estimated Difference in Means	Confidence Interval For the True Mean	P-Value
Before	-0.3375	[-0.6066, -0.0684]	**0.0156**

During	-0.3278	[-0.6181, -0.0374]	**0.0282**

After	-0.1964	[-0.3875, -0.0054]	**0.0443**

As seen in Figure 11, mean value of seg_mean medians is higher at the "during angiography" phase for both groups. This observation is in-line with the finding obtained for other features (physiological signal), which confirms that there is an increased SNS activity during the operation. Also estimated difference in mean value obtained for "before-during" comparison (Table 10) support the expectation that waiting for angiography increases the intensity of the psychophysiological responses. The estimated differences in mean for "before-during" comparison is almost ten times smaller than others for diazepam and non-diazepam group.

In Tables 11, results of the across group comparison indicate that there are statistically significant changes between different group. These observations underline the tranquilizing (SNS suppressing) influence of the diazepam. We conclude that skin conductance (GSR amplitude) and seg_mean vector are potentially good indicators to evaluate the psychophysiological responses during angiography and the influence of the tranquilizer on the psychophysiological responses.

## Discussion and Conclusion

In the studies conducted to measure psychophysiological responses, facial expressions and voice tone analyses are also performed [33]. However, these variables change very much according to culture, age and gender compared to physiological signals. For this purpose, physiological signals were used. In addition to HRV, BVP and GSR signals, there are also studies showing that psychophysiological responses may also be evaluated over electromyogram, electroencephalogram signals and pupil diameter. Therefore, new physiological signals will be included in a future study.

A recent review report that in most studies specific emotions like fear, anger, anxiety, embarrassment etc. are utilized as stimulants, in order to evaluate emotional responses that cause changes in the ANS function [34]. However, in this study, a non-invasive test which is encountered in a real-life situation and that comprises anxiety and fear [5-9] was evaluated in terms of psychophysiological responses.

All features obtained for the two groups show that SNS was more active during angiography. Making this result more comprehensible is only possible by increasing the number of subjects participating in the study. Since the results obtained for DN1/DN2 and GSR signal are statistically significant, they are the most fundamental indicators that have to be added to the studies in which psychophysiological responses are examined.

The activities of the sweat glands increase until the glands reach saturation. After an adequate resting period, in the case of re-stimulation, sweat glands will be emptied again. A new feature, like RR interval, can be computed using GSR signals. This feature should contain the knowledge of the deviations as a consequence of emptying of the sweat glands until saturation and then being emptied again with a new stimulus. Therefore, stimulation frequencies of the sweat glands were tried to be obtained by calculating the seg_mean. A problem for the seg_mean is that unlike RR intervals, sweat glands cannot produce psychophysiological responses for every stimulus due to the resting period.

It is not clear what is the resting time of a sweat gland or inter response intervals [35]. Therefore, it is not clear how long the seg_mean should be so as to capture psychophysiological responses. In fact, if resting time for sweat glands of a person can be determined, it will be possible to understand whether there is a correspondence with RR intervals or not.

The maximum sympathetic activation is anticipated during the angiography phase, while a balanced level of activity is expected between SNS and PNS at before and after angiography stages. Although waiting for angiography (at before angiography stage) causes psychophysiological responses, stimulation of the sympathetic activation in this stage will not be comparable to what it is at "during angiography" stage. Therefore, we typically anticipate a significant difference for "before-during" and "during-after" stage comparisons. In accordance with this expectation, we would like to identify a signal and/or feature that exhibit this characteristic as a potential indicator for quantification of the psychophysiological responses during angiography. Similarly, a good indicator signal/feature should not produce any statistically significant difference for "before-after" comparison. As for the analysis across the group dimension (i.e., diazepam vs. non-diazepam comparisons at three angiography stages), a good indicator may yield statistically significant differences at any stage. However, in terms of being significant, comparison results at before and after stages should be in agreement. Other combinations of results may be attributed to the different action/influence of diazepam drug at different angiography stages.

When we analyze our findings/results along these lines we conclude that the line graphs are in the same direction with the predictions. We also note that the differences are not always statistically significant. The main reason for this result may be the individual differences on the perception of and capacity to cope with stress. Therefore, maybe each person should be considered separately instead of analyzing differences across different stages and groups. We would like to address this issue in a future study.

We would like to underline following biomedical engineering contributions in our study:

1) We have recorded and analyzed several biomedical signals (ECG, BVP, GSR) at three phases of angiography (before, during, and after). This comprehensive multi-parameter signal analysis approach is "original" in terms of evaluation of psychophysiological responses. Previously, questionnaire type of "non-quantitative" techniques, which are prone to errors, were utilized mostly.

2) Further, in our study, underlying problem or cause for psychophysiological responses, i.e. coronary angiography, is of utmost clinical importance. Actually, this is the inception point of our study; basically we want to evaluate psychophysiological responses that may occur during angiography. However, while trying to address this question by designing and conducting a comprehensive clinical study, we have realized that methods used in the literature are not satisfactory. Therefore, we thought of recording relevant physiological signals at three phases: before, during, and after the angiography. Recoding of those signals during the angiography operation was a challenge by itself.

3) Features to be extracted and analyzed from the ECG signal is relatively typical; however analysis methods that we suggested and used for BVP and GSR signals are interesting and proved to be useful.

In summary, we have found out that features obtained especially from BVP and GSR signals undergo some statistically significant changes during different phases of the angiography test. Therefore, these signals and obtained features can be used in clinical studies or artificial intelligence applications where quantitative diagnosis and/or grading of psychophysiological responses are necessary.

## Competing interests

The authors declare that they have no competing interests.

## Authors' contributions

SO carried out data acquisition, data pre-processing and classification processes. MHA designed the study and prepared manuscript. MGK provided patients, performed coronary angiography and participated in the design of the study. SK participated in the design of the study and coordination. All authors read and approved the final manuscript.
